# Comparative Dermoscopic Analysis of Melanoma In Situ Versus Thin Invasive Melanoma Considering BRAF Mutational Status

**DOI:** 10.3390/jcm14186554

**Published:** 2025-09-17

**Authors:** Iulia Zboraș, Loredana Ungureanu, Simona Corina Șenilă, Orsolya Ildikó Gaál, Ștefana-Anamaria Gligor-Popa, Doinița Crișan, Sergiu Șușman, Ștefan Cristian Vesa, Rodica Cosgarea

**Affiliations:** 1 Department of Dermatology, “Iuliu Hațieganu” University of Medicine and Pharmacy, 400012 Cluj-Napoca, Romania; iuliazboras@yahoo.com (I.Z.); simonasenila@yahoo.com (S.C.Ș.); cosgarear@yahoo.com (R.C.); 2 Department of Dermatology, Emergency County Hospital, 400006 Cluj-Napoca, Romania; 3 Medical Genetics Department, “Iuliu Haţieganu” University of Medicine and Pharmacy, 400012 Cluj-Napoca, Romania; orsigaaal92@gmail.com; 4 Department of Functional Genomics and Experimental Pathology, The Oncology Institute “Prof. Dr. I. Chiricuță”, 400015 Cluj-Napoca, Romania; 5 Department of Genetics, The Oncology Institute “Prof. Dr. I. Chiricuță”, 400015 Cluj-Napoca, Romania; stefiipopa@yahoo.com; 6 Department of Pathology, “Iuliu Hațieganu” University of Medicine and Pharmacy, 400012 Cluj-Napoca, Romania; doinitacrisan@gmail.com; 7 Department of Pathology, Emergency County Hospital, 400006 Cluj-Napoca, Romania; 8 Department of Morpho-functional Sciences-Histology, “Iuliu Hațieganu” University of Medicine and Pharmacy, 400012 Cluj-Napoca, Romania; sergiu.susman@umfcluj.ro; 9 Department of Pathology-Neuropathology, IMOGEN Research Center, 400349 Cluj-Napoca, Romania; 10 Department of Pharmacology, Toxicology and Clinical Pharmacology, “Iuliu Hațieganu” University of Medicine and Pharmacy, 400337 Cluj-Napoca, Romania; stefanvesa@gmail.com

**Keywords:** melanoma, BRAF, dermoscopy

## Abstract

**Background/Objectives**: BRAF mutation is the most frequent somatic mutation in melanoma. The BRAF mutational status is crucial in selecting systemic therapy for advanced melanoma. Another important consideration is whether a melanoma is in situ or invasive. If this aspect could be known before the first surgical intervention, the appropriate surgical margins could be chosen from the beginning and a second surgical step could be avoided. Could the dermoscopic image predict the BRAF mutational status? Could it also predict if a melanoma is in situ or invasive? **Methods**: This retrospective study included 50 patients with 52 melanomas. The mutational status of the BRAF gene was determined, and the dermoscopic images were analysed. **Results**: There were no statistically significant differences between the BRAF-mutant melanoma group and the BRAF wild-type melanoma group. However, there were statistically significant differences between the dermoscopic images of melanomas in situ and thin invasive melanomas (≤1 mm Breslow thickness). Irregular dots or globules (*p* = 0.008), a blue-white veil (*p* = 0.011), milky red areas (*p* = 0.008), dotted vessels (*p* = 0.04), and linear irregular vessels (*p* = 0.016) were all more frequently present in thin invasive melanomas compared to melanomas in situ. **Conclusions**: Dermoscopy could predict whether a melanoma is in situ or invasive, but it could not predict the mutational BRAF status in the present study.

## 1. Introduction

The most frequent somatic mutation in melanoma is in the BRAF gene. BRAF mutations are present in approximately 40–60% of all melanomas, with BRAF^V600E^ being the most frequent, but there are percentage differences between different studies according to the population studied or the detection method used [1,2,3,4]. There are clinical differences between BRAF-mutant and BRAF wild-type (BRAF^WT^) melanomas. BRAF-mutant melanomas appear more frequently in younger patients, under 40 years old, while BRAF^WT^ melanomas are more commonly diagnosed in older patients [5]. Moreover, in patients under 40 years of age, the presence of the BRAF mutation was associated with a higher body mass index (BMI). The most frequent mutation in melanomas in younger patients is BRAF^V600E^, while BRAF^V600K^ is present in melanomas in older patients [3,5]. BRAF-mutant melanomas are more frequent on the trunk, an intermittently sun-exposed area, compared to other locations [6,7]. The higher count of melanocytic naevi and sun exposure in early life increases the risk of developing a BRAF-mutant melanoma [8]. However, the mutation is already present in melanocytic nevi and is considered a somatic driver mutation [3,9]. The mutation is found in dysplastic nevi almost as frequently as in melanomas in situ, but it is rare in lentigo maligna [3].

Moreover, there are differences in prognosis between BRAF-mutant and BRAF^WT^ melanomas. BRAF-mutant melanomas tend to be more aggressive, to grow more rapidly and develop metastases more rapidly, especially brain metastases, compared to BRAF^WT^ melanomas [10,11]. The BRAF mutational status is tested in advanced stages of melanoma due to the possibility of choosing between targeted therapy and immunotherapy in BRAF-mutant melanomas [12]. Targeted therapy induces a more rapid response, whereas immunotherapy yields a more durable response [13]. Over time, some patients develop resistance to targeted therapies; however, there is an option to switch to immunotherapy as an alternative treatment. Nevertheless, the response to immunotherapy is less efficient in patients who have first received targeted therapy [14,15,16]. Recent studies suggest that the first-line treatment in metastatic melanoma should be immunotherapy with consequent targeted therapy [17]. However, immunotherapy has more adverse effects related to toxicity, and in some surgical situations, targeted therapy is more appropriate [18]. Patients with BRAF-mutated melanomas and brain metastases have a lower overall survival compared to patients with BRAF^WT^ melanomas and brain metastases [19]. There is still debate on which systemic therapy is the best for BRAF-mutated melanomas, and further research is needed to discover what clinical parameters or biomarkers could indicate the prognosis and guide the choice of the best therapy in each case [11,20].

Some studies have described specific dermoscopic patterns in BRAF-mutant melanomas compared with those in BRAF^WT^ melanomas. Pozzobon et al. observed that the dermoscopic criterion of peppering was present more often in BRAF-mutated and NRAS-mutated melanomas compared to the wild-type. When excluding acral and facial melanomas, which have different dermoscopic patterns, the dermoscopic presence of ulceration was also statistically significantly associated with the presence of BRAF mutation in melanomas [21]. Bombonato et al. found a significant association between dermoscopic features like irregular peripheral streaks and ulceration with BRAF-mutated melanomas and between dotted vessels and wild-type melanomas [22]. Armengot-Carbó et al. found a significant association between BRAF-mutated melanomas and blue-white veil in dermoscopy. Patients with BRAF-mutated melanomas were younger than those with wild-type melanomas [23]. Gouillon et al. observed peppering and blue-white veil more frequently in BRAF- and NRAS-mutated melanomas compared to wild-type melanomas. At the same time, pseudopods and radial projections were more frequently found only in BRAF-mutated melanomas [24]. In contrast, Fargnoli et al. found no statistically significant differences regarding the dermoscopic features in BRAF^WT^ compared to BRAF-mutated melanomas [25].

Recently, some researchers have attempted to investigate whether dermoscopic analysis can determine if a melanoma is invasive or not and to predict Breslow thickness. Some studies described dermoscopic features that are more frequent in invasive melanomas compared to melanomas in situ [26,27,28]. Nazzaro et al. investigated which dermoscopic aspects could predict whether a melanoma is invasive among small diameter melanomas (≤5 mm). The blue-white veil and negative pigment network were associated with invasiveness [29]. Another study investigating the dermoscopic differences between melanoma in situ and invasive melanoma among small diameter melanomas observed that atypical network, irregular dots and globules and irregular hyperpigmented areas were more frequent in the group of invasive melanomas [30]. Thin melanomas and early melanomas should also be differentiated from atypical nevi using dermoscopy. Tognetti et al. proposed an algorithm (iDScore 2021) composed of 9 variables and 4 dermoscopic features to differentiate between atypical nevi and early melanomas. The included dermoscopic features are blue white veil, irregular streaks, irregular dots and globules and blue-grey peppering [31].

If it was possible to discriminate between melanomas in situ and invasive melanomas, the appropriate surgical margins could be chosen from the outset, and melanomas in situ could be directly excised with a 5 mm margin, thereby avoiding a second surgical step. In contrast, dermoscopic features associated with higher invasiveness might prioritise rapid excision. In some cases of invasive melanoma, a sentinel lymph node biopsy is recommended, and the initial surgical margins should be minimal. There are also differences regarding therapy. While melanomas in situ necessitate only surgery, invasive melanomas also require systemic therapy in more advanced cases, depending on the histopathological stage.

The aim of this study was to investigate whether there are dermoscopic differences between BRAF-mutated and BRAF^WT^ melanomas and whether dermoscopy can help differentiate between melanomas in situ (stage 0) and invasive thin melanomas (Breslow index ≤ 1 mm).

## 2. Materials and Methods

### 2.1. Study Design and Patients

The study included 50 patients with 52 melanomas diagnosed in the Department of Dermatology and Venereology at Cluj-Napoca Emergency County Hospital and CosMedica Medical Centre Cluj-Napoca between 2012 and 2020. The study was approved by the Ethics Committee of “Iuliu Hatieganu” University of Medicine and Pharmacy, Cluj-Napoca, Romania. All participants gave their informed consent before starting the study. The slides from each case were reviewed by a pathologist (D.C), and the formalin-fixed paraffin-embedded block containing more tumour tissue was selected. We included adults (≥18 years) with histopathologically confirmed melanoma, who had an available formalin-fixed paraffin-embedded (FFPE) block with sufficient material for molecular testing and corresponding dermoscopic images. We excluded all facial and acral lesions due to different dermoscopic patterns, patients < 18 years, cases without sufficient FFPE tissue for the molecular analysis, cases without a clear dermoscopic image and cases with a missing informed consent. The data collected included demographics (age, gender), family and personal history of melanoma, melanoma-specific characteristics (localisation, Breslow index, histopathological subtype and stage, mitotic rate, growth phase), other histopathological characteristics like the presence/absence of ulceration, regression, perineural invasion, lympho-vascular infiltration, pre-existent nevus, satellite micronodules and status of sentinel lymph node if performed.

### 2.2. DNA Extraction from Formalin-Fixed Paraffin-Embedded Tissues and BRAF Testing

For the analysis of BRAF mutational status, archived formalin-fixed paraffin-embedded (FFPE) tissue blocks containing tumour material were utilised. 10 µm sections were cut from each FFPE block and transferred to microcentrifuge Eppendorf tubes for DNA extraction. Genomic DNA was isolated using either the PureLink Genomic DNA Mini Kit (Thermo Fisher Scientific, Van Allen Way, Carlsbad, CA, USA) or the QIAamp DNA Micro Kit (Qiagen, Hilden, Germany), following the manufacturers’ protocols. BRAF mutation analysis was performed using the B-Raf V600E/K/D/R/M Mutation Analysis Kit for Real-Time PCR (Entrogen Inc., Woodland Hills, CA, USA), which enables detection of five point mutations at codon 600 (V600E, V600K, V600D, V600R, V600M) with a sensitivity of 1%. Amplification and detection were carried out by real-time polymerase chain reaction (RT-PCR) on a Roche LightCycler^®^ instrument (Roche Diagnostics, Mannheim, Germany), and results were analysed and interpreted in accordance with the manufacturer’s instructions.

### 2.3. Dermoscopic Analysis

**Figure A1:**
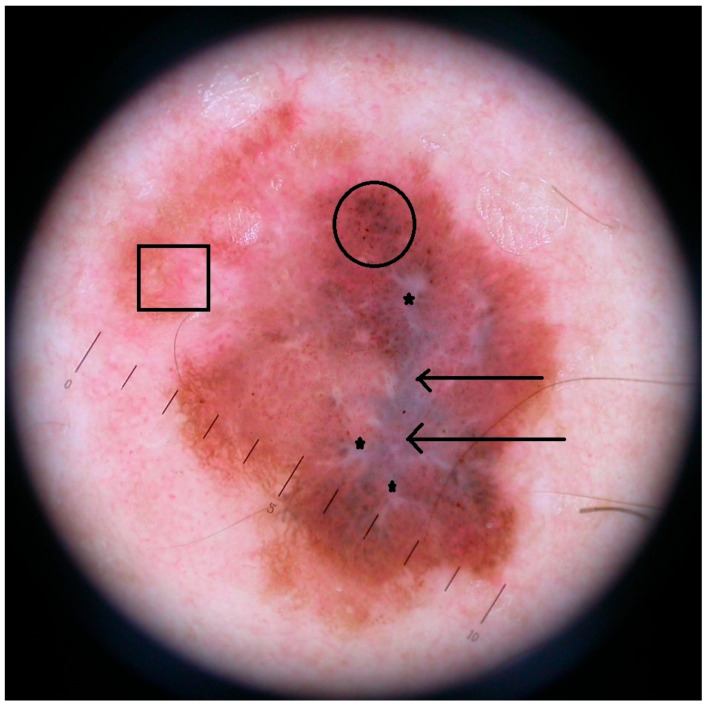
Superficial spreading melanoma, Breslow index 0.6 mm, dermoscopic features: irregular dots and globules (circle ○), blue-white veil (arrows ←), linear irregular vessels (rectangle □), shiny white structures (stars *).

**Figure A2:**
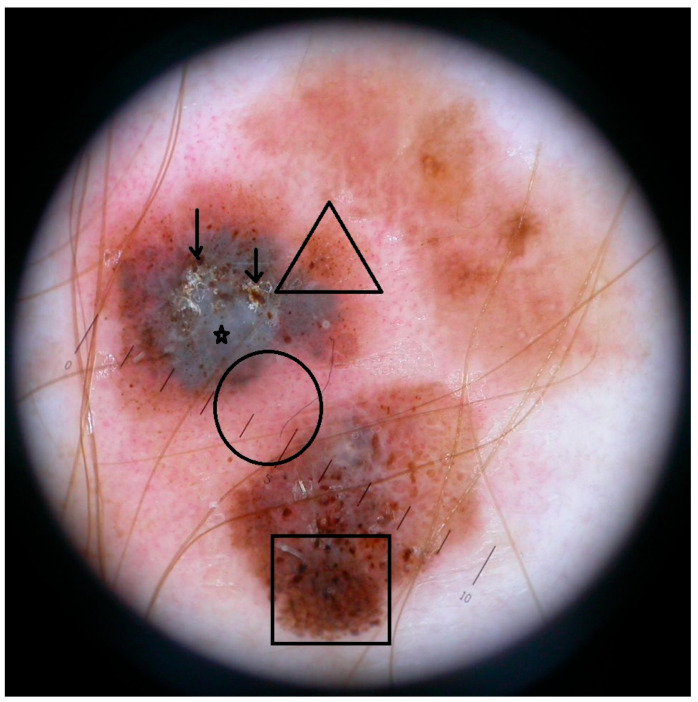
Superficial spreading melanoma, Breslow index 0.8 mm, dermoscopic features: atypical pigment network (square □), irregular dots and globules (triangle ∆), blue-white veil (star *), dermoscopic ulceration (arrows ↓), dotted vessels (circle ○).

**Figure A3:**
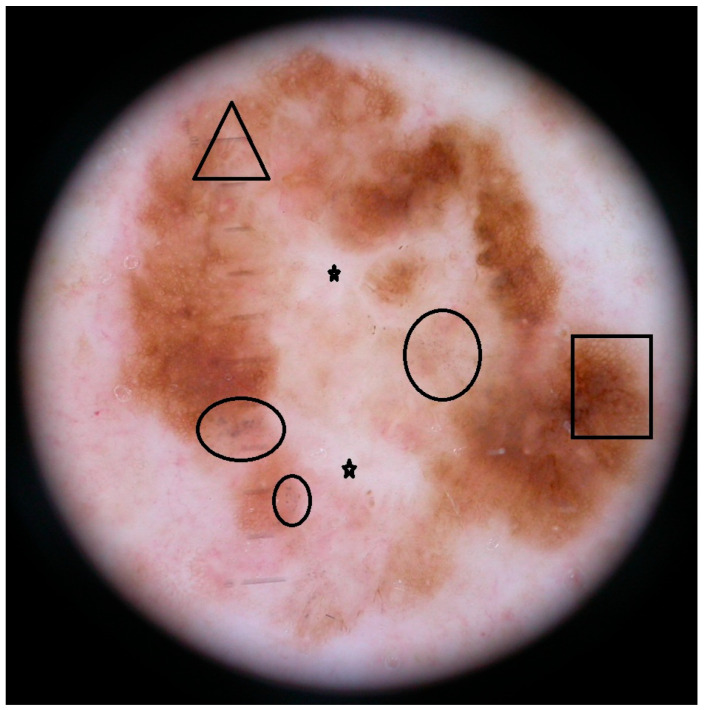
Superficial spreading melanoma in situ, dermoscopic features: atypical pigment network (rectangle □), negative network (triangle ∆), white scar-like area (stars *), peppering (circle ○).

**Figure A4:**
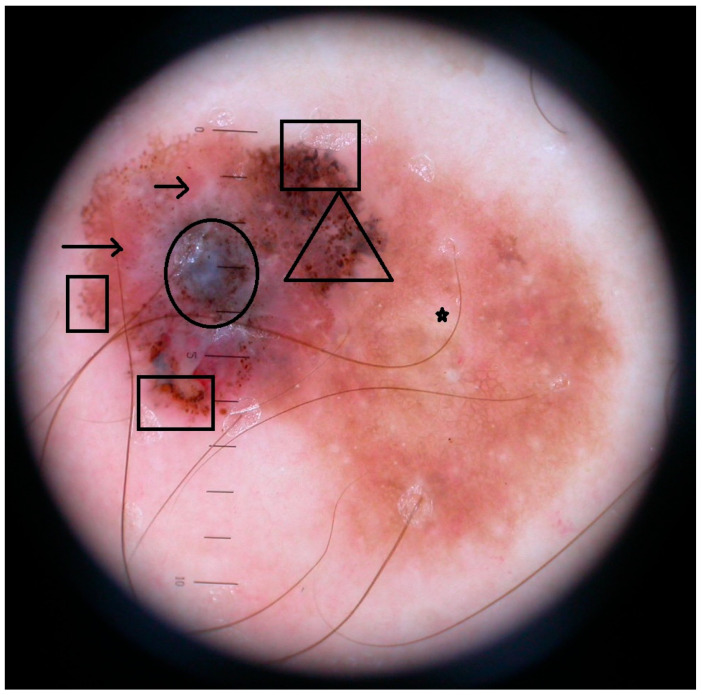
Superficial spreading melanoma, Breslow index 0.4 mm, dermoscopic features: irregular dots and globules (triangle ∆), hypopigmented structureless area (star *), irregular peripheral streaks (rectangles □), milky-red areas (arrows →), blue-white veil (circle ○).

**Figure A5:**
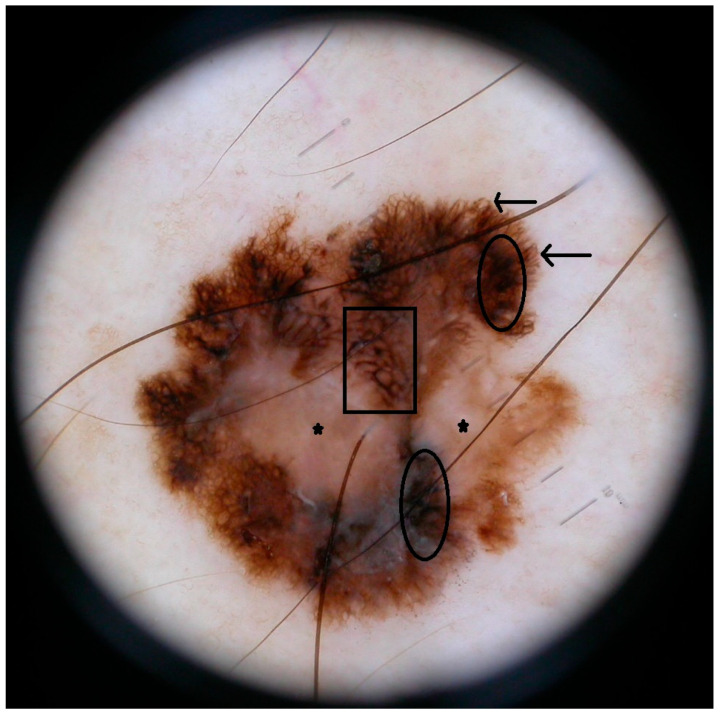
Superficial spreading melanoma in situ, dermoscopic features: irregular peripheral streaks (arrows ←), polygons/angulated lines (rectangle □), irregular hyperpigmented areas (circles ○), hypopigmented structureless areas (stars *).

**Figure A6:**
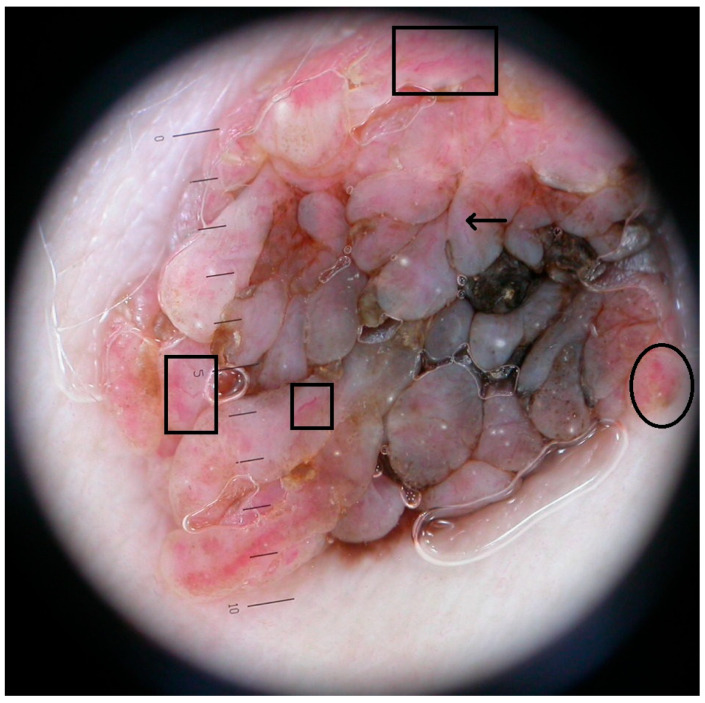
Superficial spreading melanoma developed on a preexisting nevus, Breslow index 0.5 mm, dermoscopic features: polymorphous vessels, compound of dotted vessels and glomerular vessels (circle ○), linear irregular vessels (rectangles □), corkscrew vessel (arrow ←).

Dermoscopic images were taken using a Delta 20T dermatoscope by HEINE Optotechnik, Gilching, Germany, connected to a DSLR camera via the Heine photo adapter, and then digitally stored. The images were analysed by two board-certified dermatologists, I.Z. and R.C., who were blinded to patient identity, clinical and histopathological information, as well as mutational status. The analysis comprised 16 dermoscopic features (Appendix A, Table A1 and Figure A1, Figure A2, Figure A3, Figure A4, Figure A5 and Figure A6). In the final analysis, a dermoscopic feature was considered present if both observers agreed.

### 2.4. Statistical Analysis

Statistical analysis was performed using MedCalc^®^ Statistical Software version 23.2.8 (MedCalc Software Ltd., Ostend, Belgium; https://www.medcalc.org (accessed on 22 June 2025). Qualitative data were expressed as frequency and percentage. Quantitative data were characterized by median and the 25th and 75th percentiles (non-normal distribution at Shapiro–Wilk test). Inter-rater reliability between two examiners was assessed using the Cohen’s kappa coefficient. Comparisons between groups were conducted using the chi-square or Mann–Whitney tests, whenever appropriate. A *p* value less than 0.05 was considered statistically significant. Given the number of dermoscopic features evaluated and the study’s small sample size, no correction for multiple testing was applied. The analyses should be considered as exploratory.

## 3. Results

The study included 50 patients diagnosed with 52 thin melanomas (Breslow index < 1 mm). Two patients had two cases of melanoma included in the study. Fifty-one cases were superficial spreading melanomas (SSM), while one case was Thin Invasive Melanoma of Uncertain Metastatic Potential (THIMUMP). Eighteen patients were female, and thirty-four patients were male. The patients’ ages ranged between 26 and 85 years, with a median age of 62 years and a mean age of 57.78 years. Thirty-two melanomas were localised on the trunk, fourteen melanomas were localised on the lower limb, and six melanomas were localised on the upper limb. The Breslow thickness ranged between 0 and 1 mm with a median of 0.375 mm and a mean of 0.35 mm. BRAF mutations were present in 12 cases (23.1%), of which 11 cases were BRAF^V600E^-mutated and one case was BRAF^V600M^-mutated. The clinical and histopathological characteristics are summarised in Table 1. There was no statistically significant difference between the BRAF-mutated melanoma group and the BRAF^WT^ melanoma group regarding clinical and histopathological features. However, there was a tendency towards statistical significance (*p* = 0.087) regarding the age difference between the two groups. Patients with BRAF-mutated melanomas were younger than those with BRAF^WT^ melanomas. The median age was 63 years in the BRAF^WT^ melanoma group, whereas it was 51 years in the BRAF-mutant melanoma group.

**Figure 1:**
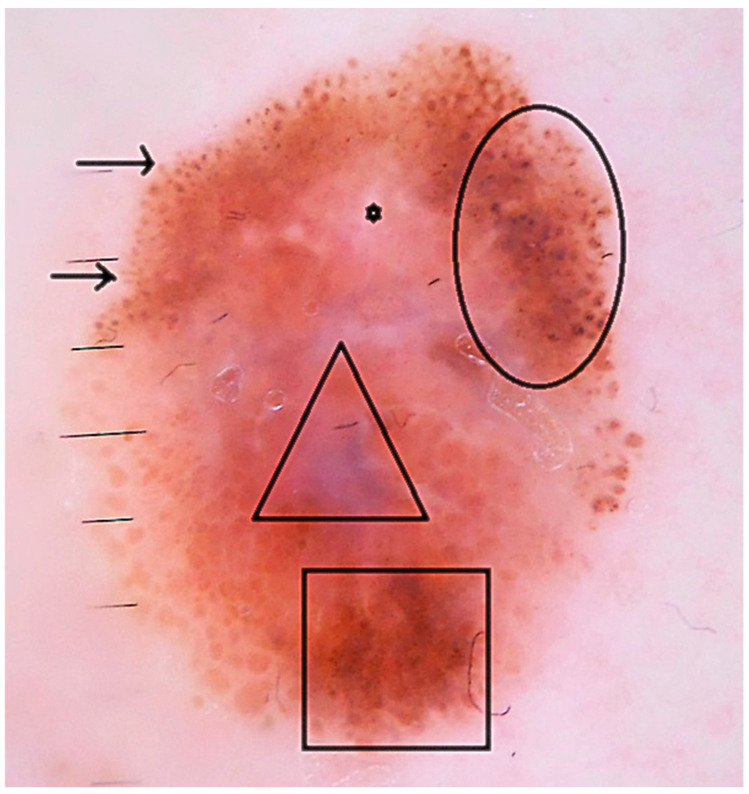
The dermoscopic image of a BRAF^V600E^ mutant melanoma that turned from pathological stage IA to stage IV in 2 years (irregular dots and globules- circle ○, irregular peripheral streaks-arrows →, white scar-like areas- star *, blue-white veil-triangle ∆, irregular hyperpigmented areas -rectangle □).

In the present study, one patient with BRAF-mutant melanoma (BRAF^V600E^), initially pT1bN0M0, clinical stage IB, pathological stage IA (AJCC 8th edition), with a Breslow index of 0.95 mm, located on the trunk, developed lymphatic and hepatic metastasis two years after the diagnosis, turning into stage IV (Figure 1).

**Figure 2:**
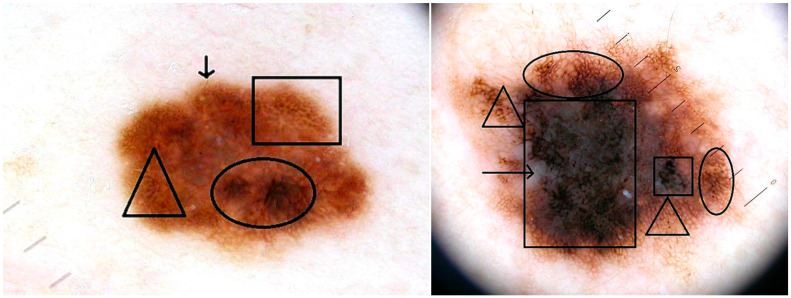
(**a**). BRAF wild-type superficial spreading melanoma in situ—Stage 0, dermoscopic features: atypical pigment network (circle ○), negative network (rectangle □), irregular dots and globules (triangle ∆), irregular peripheral streaks (arrow ↓); (**b**). BRAF V600E mutant Stage IA superficial spreading melanoma, Breslow index 0.5 mm, dermoscopic features: atypical pigment network (circle ○), irregular dots and globules (triangle ∆), blue-white veil (arrow →), irregular hyperpigmented areas in most parts of the lesion (rectangle □).

We selected 16 dermoscopic features and compared the BRAF^WT^ melanoma group with the BRAF-mutant melanoma group according to the dermoscopic images. No statistically significant differences were found between the two groups (Table 2 and Figure 2).

The present study included 22 melanomas in situ (Stage 0), considered non-invasive, and 30 Stage I (clinical stage IA/IB, pathological stage IA) melanomas, considered invasive. We used the American Joint Committee on Cancer’s (AJCC) 8th edition to classify the melanomas. The two groups were compared with respect to the previously selected dermoscopic features and found numerous statistically significant differences (Table 3). Irregular dots or globules (*p* = 0.008), blue-white veil (*p* = 0.011), milky red areas (*p* = 0.008), dotted vessels (*p* = 0.04) and linear irregular vessels (*p* = 0.016) were all more frequently present in Stage IA/B melanomas compared to Stage 0 melanomas (in situ).

## 4. Discussion

In the present study, no significant association was found between different dermoscopic features and the BRAF mutational status. Similarly to the current study, Fargnoli et al. also found no association [25]. However, other studies have found statistically significant results, although they identified different aspects as more frequent in BRAF-mutated melanomas compared to BRAF-wild-type melanomas [8,9,10,11]. Blue-white veil was more frequently observed in BRAF-mutated melanomas [23,24]. Irregular peripheral streaks, which include pseudopods and radial projections, were observed more frequently in melanomas with BRAF mutations [22,24]. Dermoscopic ulceration was also observed to be more frequent in BRAF-mutated melanomas [21,22]. When including other mitogen-activated protein kinase (MAPK) pathway mutations, peppering was observed to be more frequent in BRAF- or NRAS-mutated melanomas [21,24]. However, most studies included advanced cases of melanomas with a higher Breslow index compared to the current study, because the clinical recommendation for BRAF testing in melanoma is for stages III-IV and should be considered in stages IIB-IIC [32]. The BRAF mutational status is less important in patients with stage 0 and stage I melanomas because these patients typically do not undergo systemic therapy. In the present study, patients with BRAF-mutated melanomas were younger than those with BRAF^WT^ melanomas, a finding consistent with other studies [5,23].

When comparing dermoscopic images from in situ melanomas with thin invasive melanomas, the findings showed that irregular dots or globules, a blue-white veil, milky red areas, dotted vessels, and linear irregular vessels were statistically significantly more frequent in thin invasive melanomas. Similar results have also been reported in other studies published in this field. A study that included only thin melanomas, similar to the present study, observed that invasive melanomas have more frequent blue-white veil, milky red areas, irregular streaks and irregular network compared to melanomas in situ [33]. Another survey by Lallas et al. investigated the dermoscopic features in thin melanomas and observed that the multicomponent dermoscopic global pattern and blue-white veil were more frequent in the group of invasive melanomas. In contrast, extensive regression was an indicator of melanoma in situ [34]. Another study by Rodríguez-Lomba et al. that compared melanomas with a Breslow index < 0.8 mm and melanomas with a Breslow index ≥ 0.8 mm observed that atypical pigment network, regression and hypopigmented areas were more frequent in the first group, whereas blue-white veil, shiny white streaks, irregular vessels, blue-black pigmentation, milky red areas, rainbow pattern, pseudolacunae and ulceration were more frequent in the second group [35]. Another recent study comparing dermoscopic images of in situ melanomas, thin invasive melanomas (<1 mm Breslow index), and thick invasive melanomas (≥1 mm Breslow index) revealed differences between the three groups. When comparing melanomas in situ with invasive melanomas, some dermoscopic features were more frequent in the invasive melanoma group: atypical vessels, blue-white veil, shiny white structures and white scar-like areas. Additionally, when comparing the thin invasive melanoma group with the thick invasive melanoma group, they observed that the dermoscopic aspects of the blue-white veil and radial streaming were more frequent in the thick invasive melanoma group [36]. Another study that compared melanoma in situ, thin invasive melanoma and thick invasive melanoma observed that pseudopods (irregular peripheral streaks) and multicomponent patterns were more frequent in the group of invasive melanomas compared to melanomas in situ, atypical pigment network was more frequent in the thin invasive melanoma group and white regression structures were more frequent in the thick invasive melanoma group. However, they did not find the blue-white veil to be more frequent in the group of invasive melanomas [37]. Martínez-Piva et al. published a study that compared the same three melanoma groups as the previous one and observed blue-white veils, white shiny structures, and milky red areas more frequently in melanomas with a higher Breslow index, while angulated lines were more frequent in lesions with a lower Breslow index [38]. A recent study investigating thin melanomas found that the presence of regression structures (white patches and/or blue-grey areas), atypical vascular patterns, and the absence of a pigment network were more frequent in the group that progressed to the metastatic stage compared to those that did not [39]. Deinlein et al. observed that the dermoscopic features of the blue-white veil, milky red areas, and shiny-white streaks have been associated with the histologic description of ulceration and a mitotic rate greater than 1 mm. Moreover, in the same study, these aspects have been linked to the presence of distant metastases [40]. Some dermoscopic structures, such as the blue-white veil, milky red areas, atypical vessels, irregular peripheral streaks, shiny white streaks, and white scar-like structures, were consistently found in multiple studies, predominantly in invasive melanomas [33,35,36]. The first three dermoscopic structures were also found more frequently in the current study in thin invasive melanomas compared to melanomas in situ. Additionally, irregular dots and globules were found; however, this feature was not observed more frequently in other studies in invasive melanomas compared to melanomas in situ. Histopathologically, the blue-white veil corresponds to heavily pigmented atypical melanocytes and/or melanophages in the dermis, accompanied by an acanthotic epidermis and compact orthokeratosis [41,42]. The milky red areas correspond to an area with increased vascularity [43]. Atypical vessels (dotted vessels and linear irregular vessels) are a correlate of increased vessels in the superficial dermis [44]. Irregular peripheral streaks, also called pseudopods, correlate histopathologically with peripheral, confluent and strongly pigmented junctional nests of melanocytes or nests of melanocytes in the papillary dermis, that form tubules parallel to the surface of the skin [41,42]. The shiny white streaks are only visible with polarised dermoscopy and correspond histopathologically to altered collagen in the dermis [45,46]. On the other hand, the white scar-like structures correspond to areas of fibrosis with thickened collagen fibres in the dermis [42].

Dermoscopy can help in predicting whether a melanoma is in situ or invasive, but there no validated criteria for distinguishing between the two groups have been established. Further studies are necessary in this field to confirm the findings and identify the requirements that help differentiate between the two groups. Dermoscopy could be combined with other methods like high-frequency ultrasound and reflectance confocal microscopy, to better determine the nature of invasiveness and Breslow thickness [47,48,49].

From the series of melanomas in the present study, one patient with a BRAF-mutant melanoma developed lymphatic and hepatic metastasis two years after the initial diagnosis. While one case could be coincidental, the BRAF mutational status might serve as a prognostic factor. A meta-analysis observed that the presence of BRAF mutations was associated with poorer prognosis and overall survival in melanoma patients, although there was high heterogeneity between the studies [50].

Artificial intelligence plays an important role nowadays in every field, including dermoscopy. Several algorithms have been developed and compared with human expertise. However, the best results have been achieved when the two, artificial intelligence and humans, were combined [51,52,53,54]. Rodriguez et al. investigated whether artificial intelligence could differentiate between melanomas in situ and invasive melanomas, respectively, between melanomas with a Breslow index lower than 0.8 mm and melanomas with a Breslow index ≥ 0.8 mm. The results were promising, and artificial intelligence could support the dermatologists’ decisions in the future [55]. Another study conducted by Polesie et al. compared whether artificial intelligence or a dermatologist can differentiate better between melanomas in situ and invasive melanomas. Dermatologists outperformed artificial intelligence, but the algorithm was trained and validated on a small cohort of cases [56]. Moreover, Verdehelo et al. investigated whether Deep Learning, a type of Artificial Intelligence, can predict the BRAF mutational status from the analysis of dermoscopic images, and the results were promising [57]. With future improvements, artificial intelligence could aid dermatologists, especially those with less experience.

The limitations of the current study included the relatively small patient cohort and the low percentage of BRAF-positive cases. More dermoscopic features and scores could have been examined. The strong points of the present study are that it managed to identify significant differences between melanomas in situ and thin invasive melanomas. However, the small sample size reduced the statistical power and increased the risk of type I error. For this reason, no correction for multiple testing was made, and we consider the findings exploratory. The result should be considered important for future, larger studies. The relatively small sample size, and particularly the low proportion of BRAF-mutant melanomas, limited the statistical power to conduct multivariable analyses or calculate reliable diagnostic performance metrics (AUC, sensitivity, specificity). Further research is needed to validate and clarify these findings.

## 5. Conclusions

There were no statistically significant differences in dermoscopic analysis between BRAF-mutant and BRAF^WT^ melanomas in the present study. However, there were more significant differences between the dermoscopic features of melanomas in situ and thin invasive melanomas. These findings can be applied in clinical practice; however, further larger studies are necessary to validate them. This was a pilot study in this field, which necessitates supplementary criteria, but it represents a starting point for other larger studies.

## Figures and Tables

**Table 1:** Clinical and histopathological features by mutational status of BRAF.

Characteristics	Category	BRAF^WT^, n (%)– 40 (76.9%)	BRAF Mutated (V600E or V600M), n (%)– 12 (23.1%)	Total, n (%)– 52 (100%)	*p*-Value
Gender	F	14 (35%)	4 (33.3%)	18 (34.6%)	1.000
	M	26 (65%)	8 (66.7%)	34 (65.4%)	
Age median		63 (52; 68)	51 (41.7; 63)	62 (45.5; 68)	0.087
Family history of melanoma	No	38 (95%)	12 (100.0%)	50 (96.2%)	1.000
	Yes	2 (5%)	0 (0.0%)	2 (3.8%)	
Personal history of melanoma	No	33 (82.5%)	11 (91.7%)	44 (84.6%)	0.663
	Yes	7 (17.5%)	1 (8.3%)	8 (15.4%)	
Localisation	Trunk	24 (60%)	8 (66.6%)	32 (61.5%)	0.892
	Lower limb	11 (27.5%)	3 (25%)	14 (27%)	
	Upper limb	5 (12.5%)	1 (8.3%)	6 (11.5%)	
Breslow index median		0.3 (0; 0.6)	0.55 (0; 0.85)	0.375 (0; 0.62)	0.206
pT AJCC 8th edition	Tis	18 (45%)	4 (33.3%)	22 (42.3%)	0.690
	pT1a	16 (40%)	5 (41.7%)	21 (40.4%)	
	pT1b	6 (15%)	3 (25%)	9 (17.3%)	
Clinical TNM staging AJCC 8th edition	0	18 (45%)	4 (33.3%)	22 (42.3%)	0.690
	IA	16 (40%)	5 (41.7%)	21 (40.4%)	
	IB	6 (15%)	3 (25%)	9 (17.3%)	
Pathological TNM staging AJCC 8th edition	0	18 (45%)	4 (33.3%)	22 (42.3%)	0.526
	IA	22 (55%)	8 (66.7%)	30 (57.7%)	
Ulceration	No	37 (92.5%)	12 (100.0%)	49 (94.2%)	1.000
	Yes	3 (7.5%)	0 (0.0%)	3 (5.8%)	
Regression	No	27 (67.5%)	9 (75.0%)	36 (69.2%)	0.733
	Yes	13 (32.5%)	3 (25.0%)	16 (30.8%)	
Mitotic rate median		0 (0;1)	0.5 (0; 2.75)	0 (0;1)	0.414
Vertical growth phase	No	31 (77.5%)	9 (75.0%)	40 (76.9%)	1.000
	Yes	9 (22.5%)	3 (25.0%)	12 (23.1%)	
Pre-existent nevus	No	23 (57.5%)	9 (75.0%)	32 (61.5%)	0.330
	Yes	17 (42.5%)	3 (25.0%)	20 (38.5%)	

**Table 2:** Dermoscopic features by mutational status of BRAF.

Characteristics	Category	BRAF^WT^ n (%)–40 (77.4%)	BRAF Mutant n (%)–12 (22.6%)	Total n (%)–52 (100%)	Kappa (k)	*p*-Value
Atypical pigment network	Present	29 (72.5%)	7 (58.3%)	36 (69.2%)	0.911	0.478
	Absent	11 (27.5%)	5 (41.7%)	16 (30.8%)		
Negative network	Present	5 (12.5%)	2 (16.7%)	7 (13.5%)	1	0.656
	Absent	35 (87.5%)	10 (83.3%)	45 (86.5%)		
Irregular dots/globules	Present	31 (77.5%)	8 (66.7%)	39 (75%)	0.898	0.466
	Absent	9 (22.5%)	4 (33.3%)	13 (25%)		
Irregular peripheral streaks	Present	24 (60%)	7 (58.3%)	31 (59.6%)	0.921	1.000
	Absent	16 (40%)	5 (41.7%)	21 (40.4%)		
White, scar-like areas	Present	15 (37.5%)	5 (41.7%)	20 (38.5%)	1	1.000
	Absent	25 (62.5%)	7 (58.3%)	32 (61.5%)		
Blue grey peppering	Present	10 (25%)	3 (25.0%)	13 (25%)	1	1.000
	Absent	30 (75%)	9 (75.0%)	39 (75%)		
Blue-white veil	Present	24 (60%)	8 (66.7%)	32 (61.5%)	0.960	0.747
	Absent	16 (40%)	4 (33.3%)	20 (38.5%)		
Milky red areas	Present	15 (37.5%)	3 (25.0%)	18 (34.6%)	0.958	0.507
	Absent	25 (62.5%)	9 (75.0%)	34 (65.4%)		
Irregular hyperpigmented areas	Present	37 (92.5%)	11 (91.7%)	48 (92.3%)	0.649	1.000
	Absent	3 (7.5%)	1 (8.3%)	4 (7.7%)		
Hypopigmented structureless areas	Present	20 (50%)	3 (25.0%)	23 (44.2%)	0.961	0.188
	Absent	20 (50%)	9 (75.0%)	29 (55.8%)		
Polygons/angulated lines	Present	9 (22.5%)	2 (16.7%)	11 (21.2%)	0.892	1.000
	Absent	31 (77.5%)	10 (83.3%)	41 (78.8%)		
Ulceration	Present	1 (2.5%)	1 (8.3%)	2 (3.8%)	1	0.412
	Absent	39 (97.5%)	11 (91.7%)	50 (96.2%)		
Shiny white structures	Present	14 (35%)	4 (33.3%)	18 (34.6%)	0.959	1.000
	Absent	26 (65%)	8 (66.7%)	34 (65.4%)		
Dotted vessels	Present	12 (30%)	3 (25.0%)	15 (28.8%)	0.911	1.000
	Absent	28 (70%)	9 (75.0%)	37 (71.2%)		
Corkscrew vessels	Present	1 (2.5%)	0 (0.0%)	1 (1.9%)	1	1.000
	Absent	39 (97.5%)	12 (100.0%)	51 (98.1%)		
Linear irregular vessels	Present	6 (15%)	1 (8.3%)	7 (13.5%)	0.791	1.000
	Absent	34 (85%)	11 (91.7%)	45 (86.5%)		
Polymorphous vessels	Present	1 (2.5%)	0 (0.0%)	1 (1.9%)	0.381	1.000
	Absent	39 (97.5%)	12 (100.0%)	51 (98.1%)		

**Table 3:** Dermoscopic features by melanoma histopathological stage.

Characteristics	Category	In situ (Stage 0) n (%)–22 (42.3%)	Stage IA/IB n (%)–30 (57.7%)	Total n (%)–52 (100%)	Kappa (k)	*p*-Value
Atypical pigment network	Present	15 (68.2%)	21 (70.0%)	36 (69.2%)	0.911	1.000
	Absent	7 (31.8%)	9 (30.0%)	16 (30.8%)		
Negative network	Present	4 (18.2%)	3 (10.0%)	7 (13.5%)	1	0.438
	Absent	18 (81.8%)	27 (90.0%)	45 (86.5%)		
Irregular dots/globules	Present	12 (54.5%)	27 (90.0%)	39 (75%)	0.898	0.008
	Absent	10 (45.5%)	3 (10.0%)	13 (25%)		
Irregular peripheral streaks	Present	11 (50%)	20 (66.7%)	31 (59.6%)	0.921	0.263
	Absent	11 (50%)	10 (33.3%)	21 (40.4%)		
White, scar-like areas	Present	8 (36.4%)	12 (40.0%)	20 (38.5%)	1	1.000
	Absent	14 (63.6%)	18 (60.0%)	32 (61.5%)		
Blue grey peppering	Present	6 (27.3%)	7 (23.3%)	13 (25%)	1	0.757
	Absent	16 (72.7%)	23 (76.7%)	39 (75%)		
Blue-white veil	Present	9 (40.9%)	23 (76.7%)	32 (61.5%)	0.960	0.011
	Absent	13 (59.1%)	7 (23.3%)	20 (38.5%)		
Milky red areas	Present	3 (13.6%)	15 (50.0%)	18 (34.6%)	0.958	0.008
	Absent	19 (86.4%)	15 (50.0%)	34 (65.4%)		
Irregular hyperpigmented areas	Present	19 (86.4%)	29 (96.7%)	48 (92.3%)	0.649	0.299
	Absent	3 (13.6%)	1 (3.3%)	4 (7.7%)		
Hypopigmented structureless areas	Present	11 (50%)	12 (40.0%)	23 (44.2%)	0.961	0.576
	Absent	11 (50%)	18 (60.0%)	29 (55.8%)		
Polygons/angulated lines	Present	6 (27.3%)	5 (16.7%)	11 (21.2%)	0.892	0.495
	Absent	16 (72.7%)	25 (83.3%)	41 (78.8%)		
Ulceration	Present	0 (0%)	2 (6.7%)	2 (3.8%)	1	0.502
	Absent	22 (100%)	28 (93.3%)	50 (96.2%)		
Shiny white structures	Present	8 (36.4%)	10 (33.3%)	18 (34.6%)	0.959	1.000
	Absent	14 (63.6%)	20 (66.7%)	34 (65.4%)		
Dotted vessels	Present	3 (13.6%)	12 (40.0%)	15 (28.8%)	0.911	0.040
	Absent	19 (86.4%)	18 (60.0%)	37 (71.2%)		
Corkscrew vessels	Present	0 (0.0%)	1 (3.3%)	1 (1.9%)	1	1.000
	Absent	22 (100.0%)	29 (96.7%)	51 (98.1%)		
Linear irregular vessels	Present	0 (0.0%)	7 (23.3%)	7 (13.5%)	0.791	0.016
	Absent	22 (100.0%)	23 (76.7%)	45 (86.5%)		
Polymorphous vessels	Present	0 (0.0%)	1 (3.3%)	1 (1.9%)	0.381	1.000
	Absent	22 (100.0%)	29 (96.7%)	51 (98.1%)		

## Data Availability

The datasets used and/or analyzed during the current study are available from the corresponding author on reasonable request.

## Abbreviations

The following abbreviations are used in this manuscript:

BRAF WT	BRAF wild-type
BMI	Body mass index
FFPE	Formalin-fixed paraffin-embedded
RT-PCR	Real-time polymerase chain reaction
SSM	Superficial spreading melanoma
ALM	Acral lentiginous melanoma
THIMUMP	Thin Invasive Melanoma of Uncertain Metastatic Potential
MAPK	Mitogen-activated protein kinase
AJCC	American Joint Committee on Cancer’s

## Appendix A

The dermoscopic features analysed were: atypical pigment network, negative network, irregular dots/globules, irregular peripheral streaks, white scar-like areas, blue grey peppering, blue-white veil, milky red areas, irregular hyperpigmented areas, hypopigmented structureless areas, polygons/angulated lines, ulceration, shiny white structures, dotted vessels, corkscrew vessels, linear irregular vessels and polymorphous vessels. The definitions are exemplified in Table A1 and illustrated in the Figure A1, Figure A2, Figure A3, Figure A4, Figure A5 and Figure A6.

**Table A1:** Definitions of the selected dermoscopic features.

Dermoscopic Feature	Definition
Atypical pigment network [58]	Network with an increased variability in the colour, thickness and spacing of the lines that are asymmetrically distributed;
Negative network [58]	Broadened interconnecting hypopigmented lines that surround elongated and curvilinear globules;
Irregular dots/globules [58]	Dots and/or globules with variable colour, size, shape, or spacing distributed in an asymmetric pattern;
Irregular peripheral streaks [58]	Comprise radial streaming (radial linear extension at the periphery of the lesion) and pseudopods (bulbous and often curved projections seen at the periphery of the lesion, either directly associated with a network or solid tumour border);
White scar-like areas [58]	Area of white that is whiter than surrounding normal -appearing skin (true scarring);
Blue grey peppering [58]	Consists of blue or grey dots;
Blue-white veil [58]	An irregular blue blotch with a whitish, ground-glass haze overlay;
Milky red areas [58]	Milky-white appearance or pinkish, structureless areas (strawberry and ice cream-like), containing a red vascular blush, with no specific distinguishable vessels;
Irregular hyperpigmented areas [34]	Multiple, small, irregularly shaped and bizarrely outlined dark areas;
Hypopigmented structureless areas [42]	Areas with a lighter pigment compared to the surrounding lesion, but with the same or slightly lighter pigment compared to the surrounding normal skin;
Polygons/angulated lines [58]	Grey-brown lines that are connected at an angle and form polygons;
Ulceration [59,60]	A large structureless area with orange, dark red to brown colouration, with a serous crust
Shiny white structures [58]	Compound of shiny white streaks (former chrysalis, chrysalids, crystalline; short discrete white lines oriented parallel and perpendicular to each other, seen only under polarised dermoscopy) and shiny white blotches and strands (white structures in the form of circles, oval structures, or large structureless areas longer and less well-defined lines oriented parallel or distributed unorganized, or forming blotches -shiny white clods, seen only under polarised dermoscopy);
Dotted vessels [44,58]	Dots; tiny pinpoint vessels;
Corkscrew vessels [58]	Tightly coiled and tortuous vessels with bends twisted along a central axis;
Linear irregular vessels [44,58]	Linear, irregularly shaped, sized, and distributed vessels;
Polymorphous vessels [58]	Multiple types of vessels are present.
